# Supplementary material for: SARS-CoV-2 infects adipose tissue in a fat depot- and viral lineage-dependent manner
Source: Nat Commun. 2022 Sep 29;13:5722. doi: 10.1038/s41467-022-33218-8 (PMC9521555; doi:10.1038/s41467-022-33218-8)
Supplement: Supplementary file 1 — Supplementary Information [file 41467_2022_33218_MOESM1_ESM.pdf]

Supplementary Information

Saccon et al. SARS-CoV-2 infects adipose tissue in a fat depot- and viral lineage-dependent manner

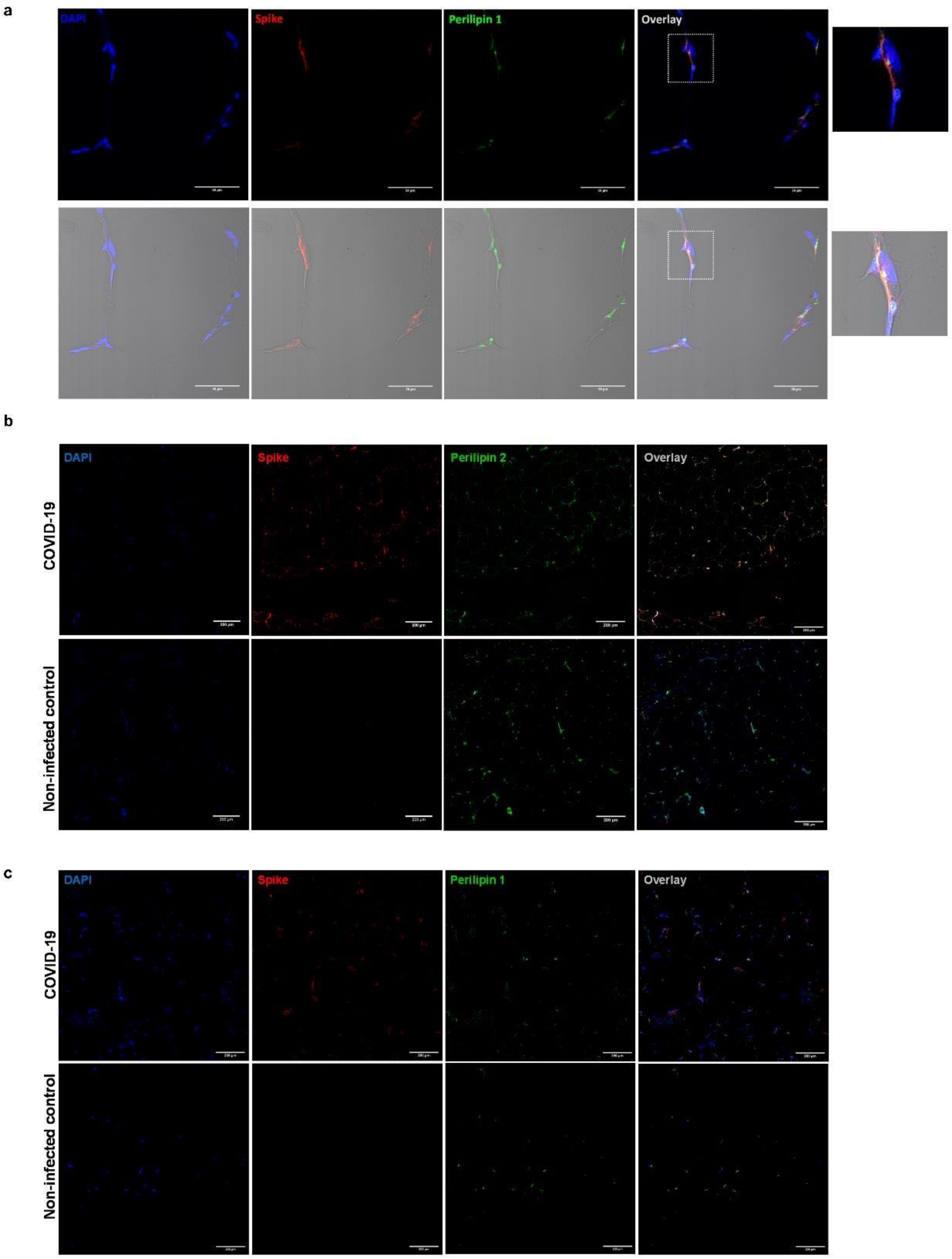

Supplementary Fig. 1. SARS-CoV-2 infects human adipose tissue.
Representative images of the immunofluorescence analysis of thoracic subcutaneous adipose tissue samples of two individuals who died of COVID-19 or one non-infected control (negative control). DAPI, blue. SARS-CoV-2 spike protein, red. Perilipin 1 or 2, green. **a** Representative image at higher magnitude of the adipose tissue of an individual with COVID-19. Scale bar = 50 µm. **b** and **c**, Representative images at lower magnitude of the adipose tissue of an individual with COVID-19 and a non-infected control, composite of 16

overlapping frames from the same individual. Scale bar = 200 μm. This experiment was repeated once with similar results. Source data are provided as a Source Data file.

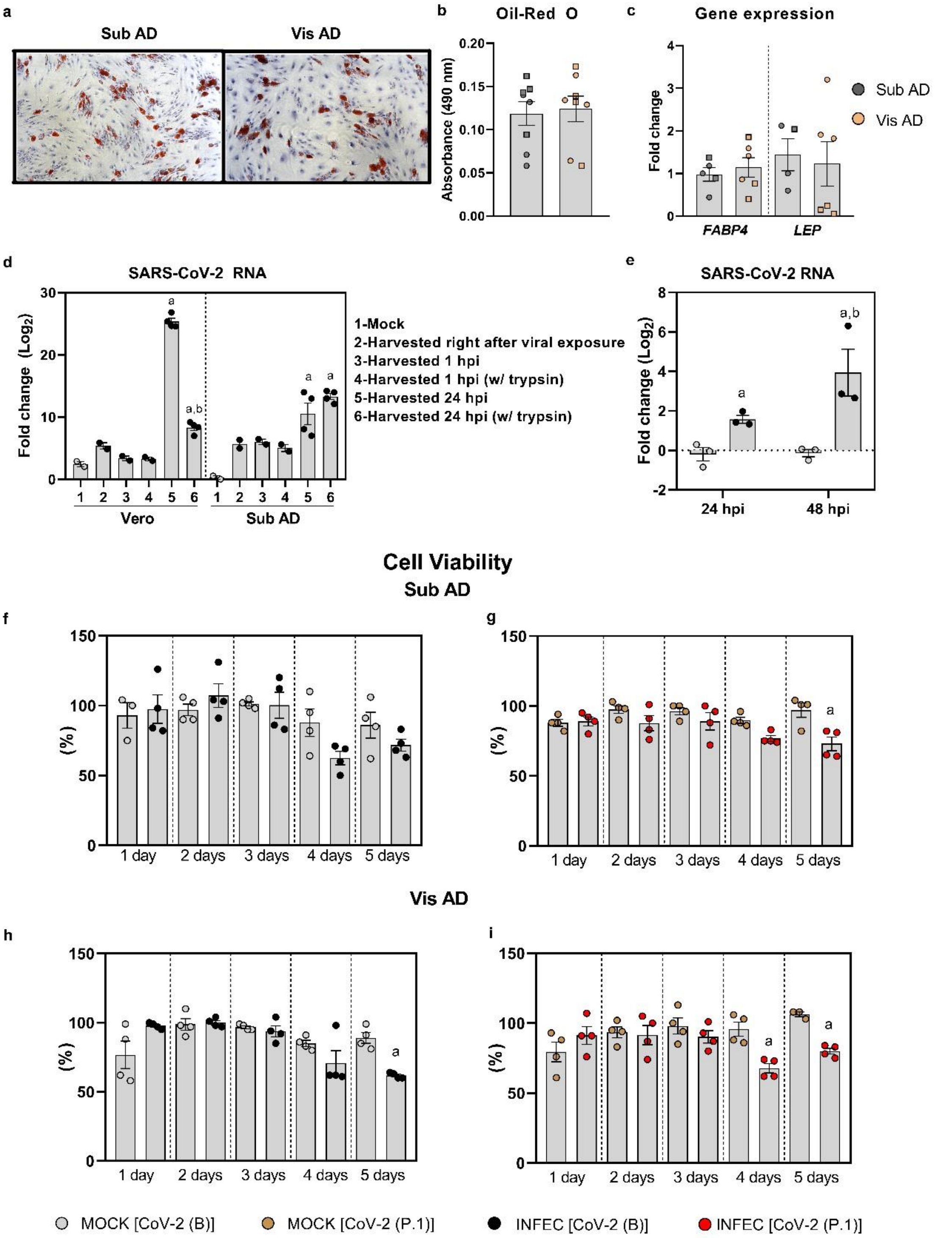

Supplementary Fig. 2. SARS-CoV-2 infects and replicates in human adipose tissue cells *in vitro*, leading to cell death. Human adipose tissue-derived stromal-vascular cells from subcutaneous and visceral fat depots were differentiated into adipocytes (Sub AD and Vis AD, respectively). **a** Representative images of cells stained with Oil Red O after adipocyte differentiation. **b** Oil Red O quantification. **c** *FABP4* and *LEP* after differentiation. **d** SARS-CoV-2 RNA in infected Sub AD cells harvested according to the indicated protocol. INFEC, infected (MOI = 1 for 1h). MOCK, inactivated virus. CoV-2(B), original B SARS-CoV-2 lineage. CoV-2(P.1), SARS-CoV-2 gamma variant P.1. hpi, hours post-infection. Cells were washed with PBS before harvesting or trypsin incubation. One-way ANOVA determined a significant effect of the conditions ($P<0.0001$). Tukey's multiple comparison test revealed differences between the groups: a, $P<0.0001$ vs. MOCK; b, $P<0.0001$ vs. 5. **e** SARS-CoV-2 RNA in infected Sub AD cells harvested 24 and 48 hpi. Two-way ANOVA was applied to determine the effect of the infection ($P<0.0001$), time ($P<0.01$) and interaction between infection x time ($P<0.01$). Tukey's multiple comparison test revealed differences between groups: a, $P<0.01$ vs. MOCK (24 hpi) and $P<0.0001$ vs. MOCK (48 hpi); b, $P<0.0001$ vs. INFEC

(24 hpi). **f-i** Cell viability. One-way ANOVA was applied to determine the effect of infection on cell viability: $P<0.01$ for Sub AD CoV-2(B) and CoV-2(P.1), and Vis AD CoV-2(P.1) or $P<0.0001$ for Vis AD CoV-2(B). Tukey's multiple comparison test revealed differences between groups: a, $P<0.05$ vs. MOCK for Vis AD CoV-2(B) or $P<0.01$ vs. MOCK for Sub AD CoV-2(P.1) and Vis AD CoV-2(P.1). Data are mean ± SEM of 2-3 independent pools of cells from 1-2 donors. Vero cells were used as reference. Different donors are distinguished by circles and squares, while independent pools of cells from the same donor (or Vero) are marked by the same symbol. Experiments were repeated at least once with similar results, with the exception of **d**, which was corroborated by other data in the manuscript, and **f-i**, although the calculated interassay variability was relatively low in these experiments, the repeated measures were consistent, controls worked as expected and biological and technical variability was accounted for in the analysis. Source data are provided as a Source Data file.

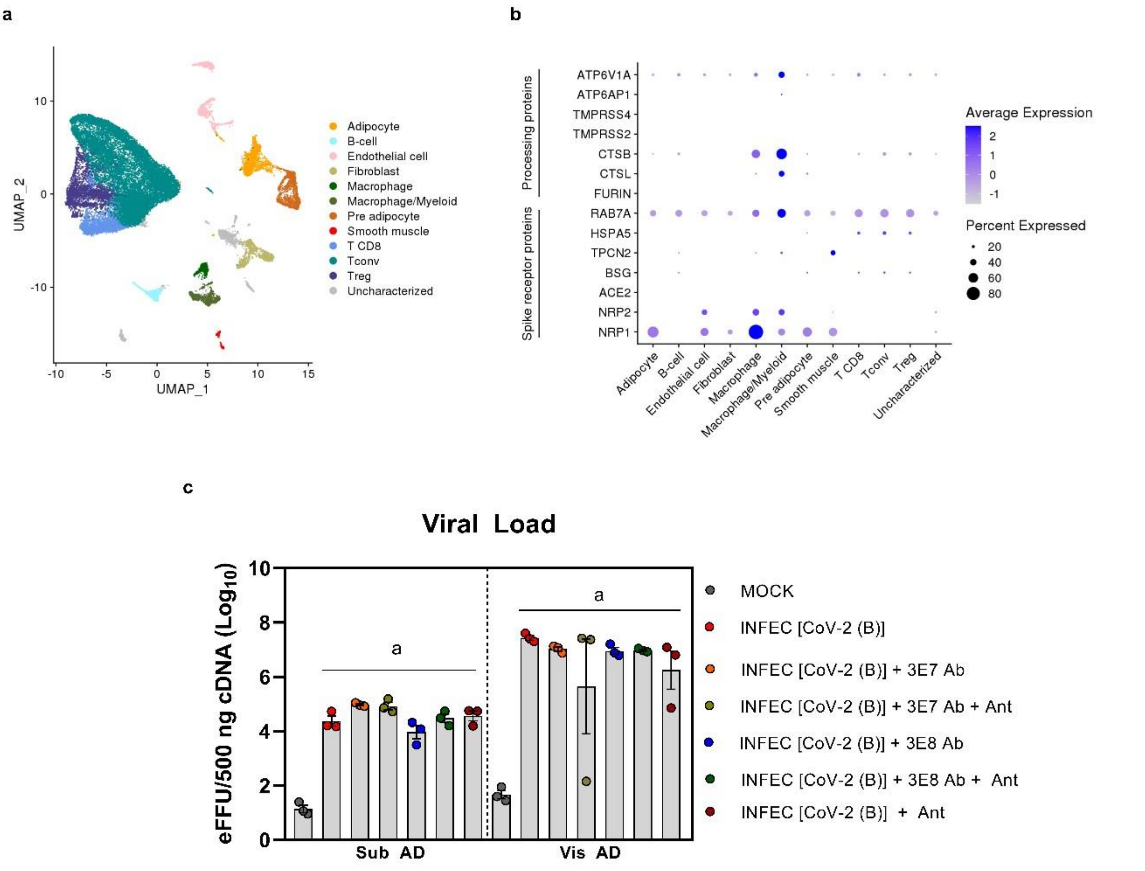

Supplementary Fig. 3. NRP1 is not required for SARS-CoV-2 entrance in human adipose tissue cells. **a** Publicly available single-nucleus RNA sequencing data of human deep-neck adipose tissue was processed and analyzed to identify single-cell population clusters that are shown in the UMAP. Cell clusters were depicted in different colors and were used to access the expression of genes within each population. Uncharacterized clusters were represented in gray. **b** Expression of genes coding for proteins described to bind SARS-CoV-2 spike protein and/or facilitate SARS-CoV-2 entry. The dot size represents the percentage of cells expressing the selected genes within a cell population and the gray-blue scale represents the average expression within the cluster. Tconv, conventional T cells. Treg, regulatory T cells. **c** Human adipose tissue-derived stromal-vascular cells isolated from subcutaneous or visceral adipose tissues were differentiated into adipocytes (Sub AD and Vis AD, respectively), preincubated with NRP1 neutralizing antibodies (E7 Ab and E8 Ab) and/or antagonist (Ant), exposed to SARS-CoV-2 [CoV-2(B) lineage, MOI = 1 for 1h] (INFECT) and harvested 24 hours post-infection for viral load quantification. MOCK, inactivated virus. eFFU, equivalent to focus forming units. Each dot represents independent pools of cells from one donor. One-way ANOVA was applied to determine the effect of the infection (P<0.0001). Tukey's multiple comparison test was performed to identify differences between the groups. These differences are depicted in letters. a, P<0.01 vs. MOCK (Sub AD) or P<0.0001 vs. MOCK (Vis AD). Data represent the mean ± SEM of experiments performed using 3 independent pools of cells from one donor. Experiments in **c** have not been repeated, although the calculated interassay variability was relatively low, the same observation was obtained using different antibodies in combination or not with an antagonist, controls worked as expected and biological and technical variability was accounted for in the analysis. Source data are provided as a Source Data file.

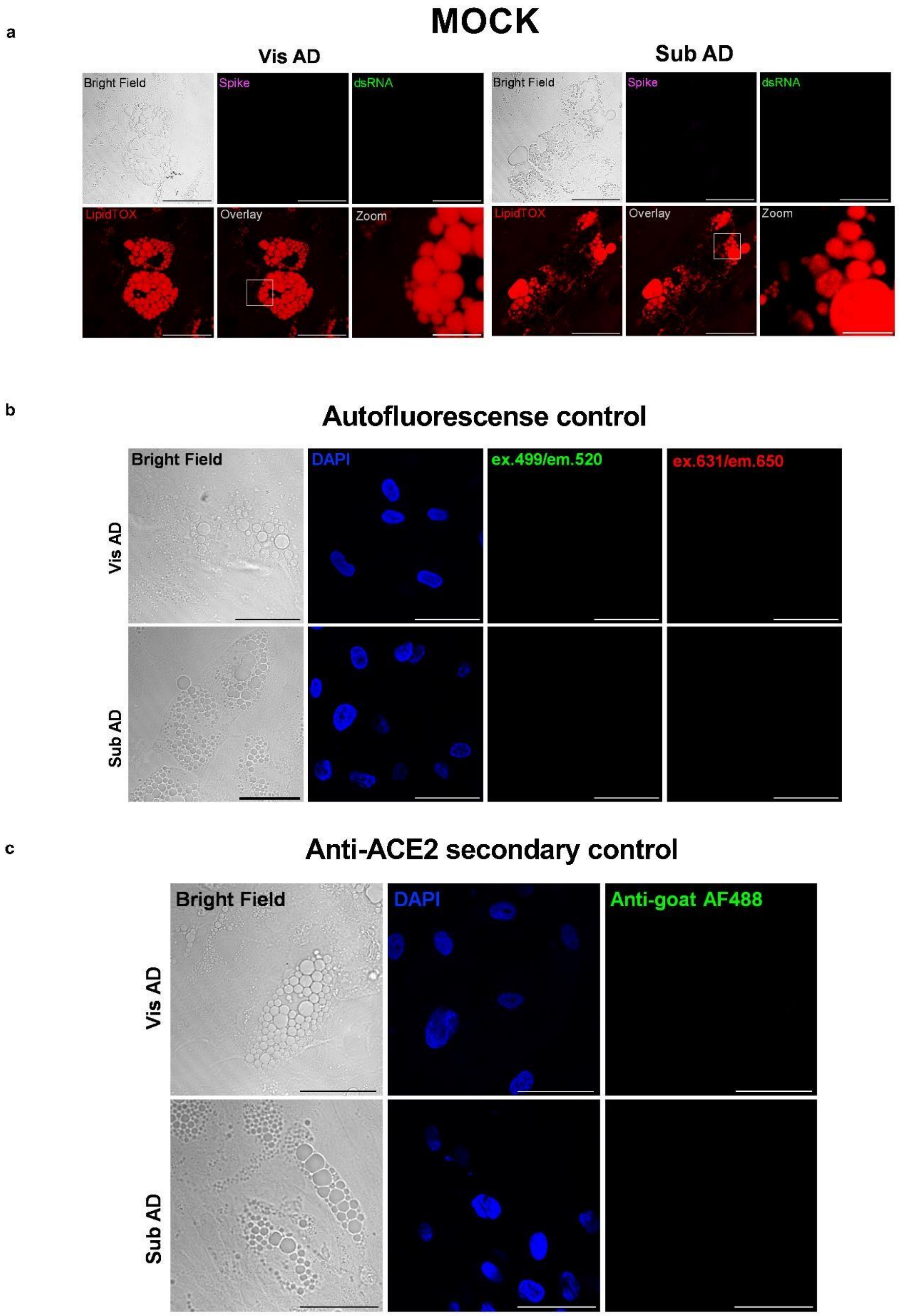

Supplementary Figure 4. Controls of immunofluorescence in human adipose tissue-derived stromal-vascular cells differentiated in adipocytes. Stromal-vascular cells from human subcutaneous and visceral fat depots were differentiated into adipocytes (Sub AD and Vis AD, respectively). Representative of 6 images from 2 independent pools of cells. **a** Mock controls. SARS-CoV-2 spike protein, magenta. Double stranded

RNA (dsRNA), green. LipidTOX, red. Scale bar = 50 µm (zoom = 10 µm). **b** Autofluorescence controls. DAPI, blue. ex.499/em.520, green. ex.631/em.650, red. Scale bar = 50 µm. **c** Secondary antibody control. DAPI, blue. anti-goat AF488 (no ACE2 primary antibody), green. Scale bar = 50 µm. These experiments are related to Figure 2b and were repeated once with similar results. Source data are provided as a Source Data file.

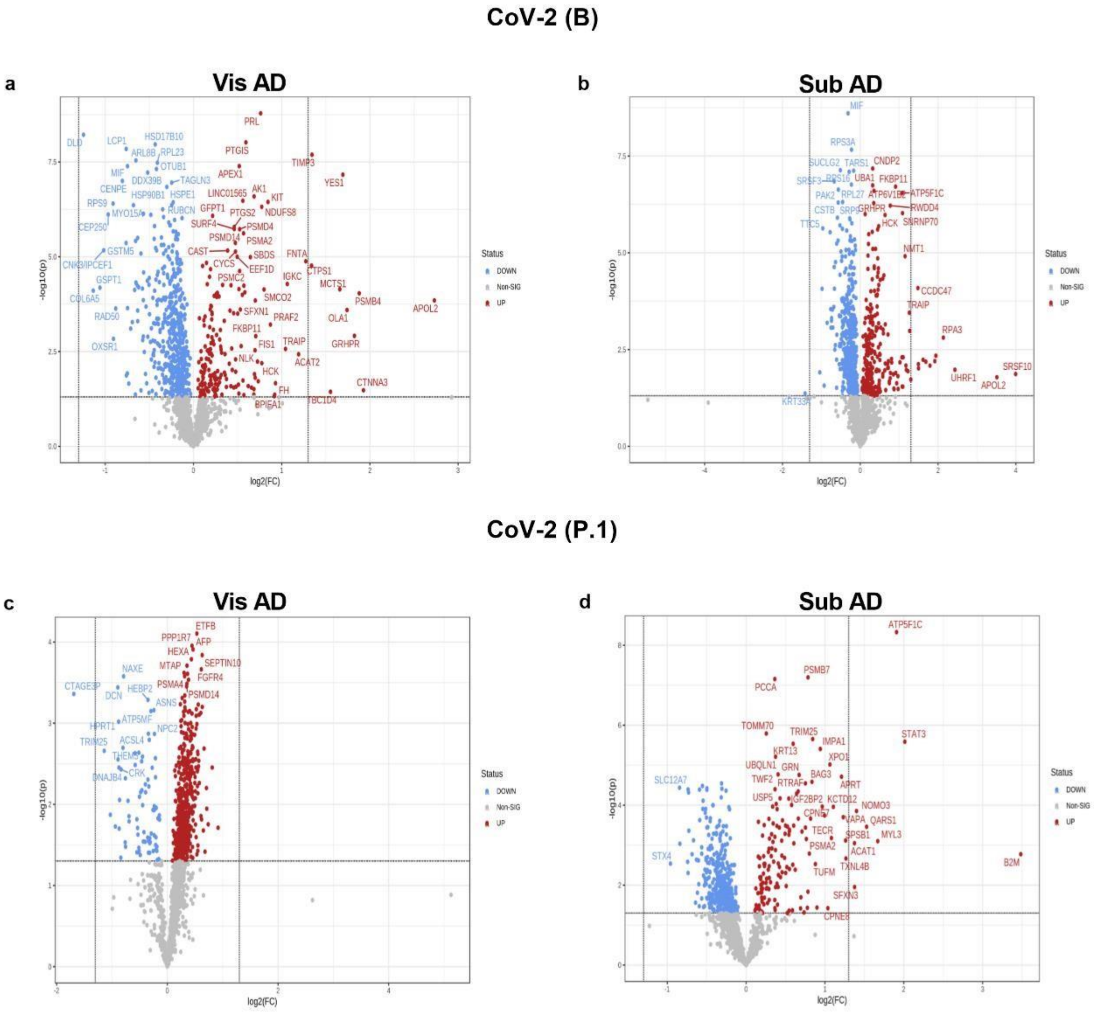

Supplementary Fig. 5. Volcano plots showing changes in the human adipose tissue cell proteome upon SARS-CoV-2 infection. a-d Human adipose tissue-derived stromal-vascular cells isolated from visceral adipose tissue and subcutaneous adipose tissue were differentiated into adipocytes (Vis AD and Sub AD, respectively), exposed to the ancient [CoV-2(B)] or the P.1 [CoV-2(P.1)] SARS-CoV-2 lineages (MOI = 1 for 1h) (INFEC) and harvested 24 hours post-infection for proteomics analysis. MOCK, inactivated virus. Log_2 of the fold change (INFECT/MOCK) in the X axis and $-Log_2$ of the P value in the Y axis. Non-SIG, non-significant. Proteomics were run using samples from 2-3 donors in technical triplicate. The mass spectrometry analysis was repeated once with similar results. Source data are provided in Supplementary Data 1.

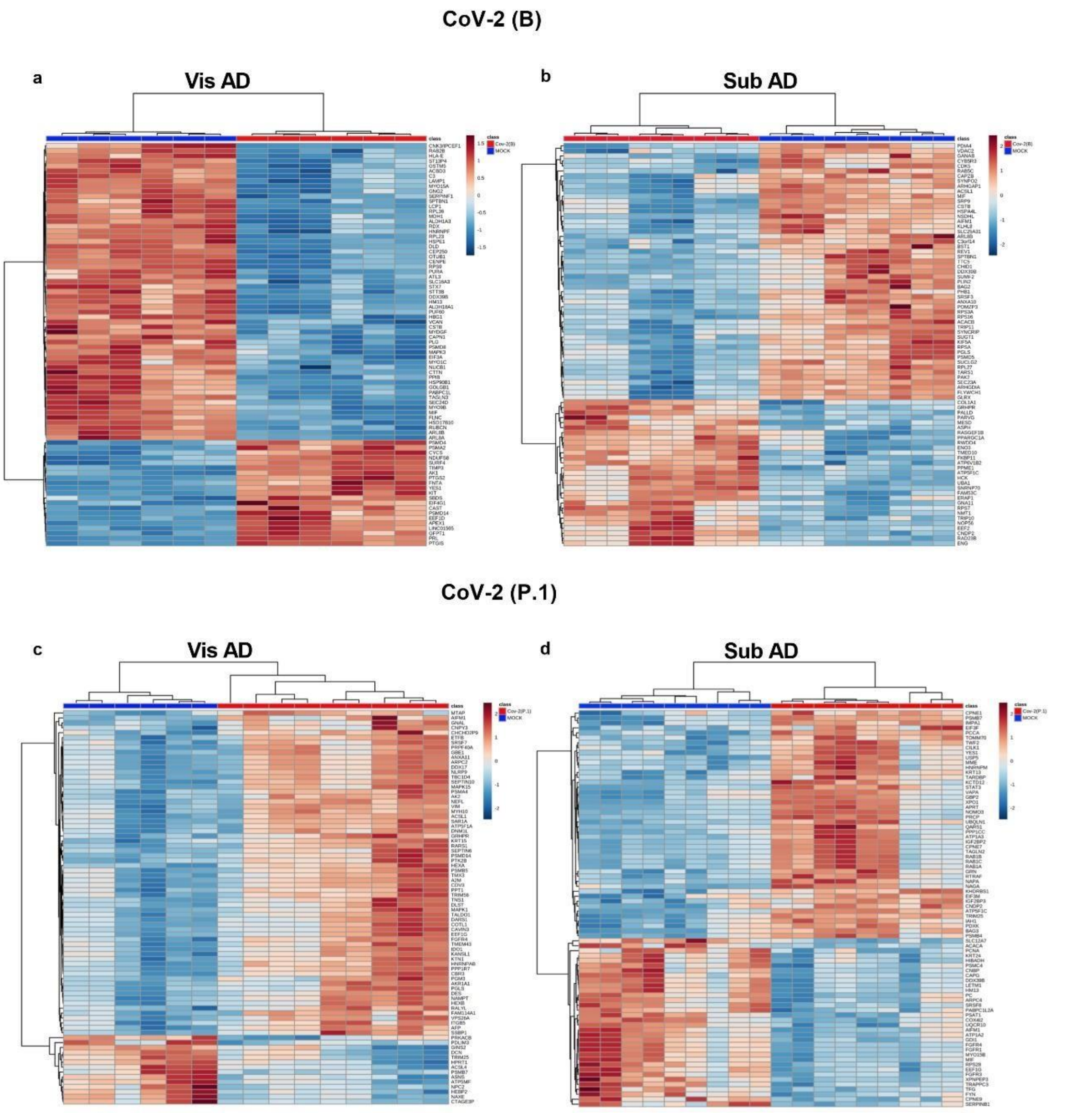

Supplementary Fig. 6. Heatmaps showing changes in the human adipose tissue cell proteome upon SARS-CoV-2 infection. a-d Human adipose tissue-derived stromal-vascular cells isolated from visceral adipose tissue and subcutaneous adipose tissue were differentiated into adipocytes (Vis AD and Sub AD, respectively), exposed to the ancient [CoV-2(B)] or the P.1 [CoV-2(P.1)] SARS-CoV-2 lineages (MOI = 1 for 1h) and harvested 24 hours post-infection for proteomics analysis. Heatmap of significantly altered proteins (q value < 0.01). Heatmap was constructed using the normalized peak intensity for each protein. Clusterization of intensities was made by average distance and Pearson's correlation. Proteomics were run using samples from 2-3 donors in technical triplicate. The mass spectrometry analysis was repeated once with similar results. Source data are provided in Supplementary Data 1.

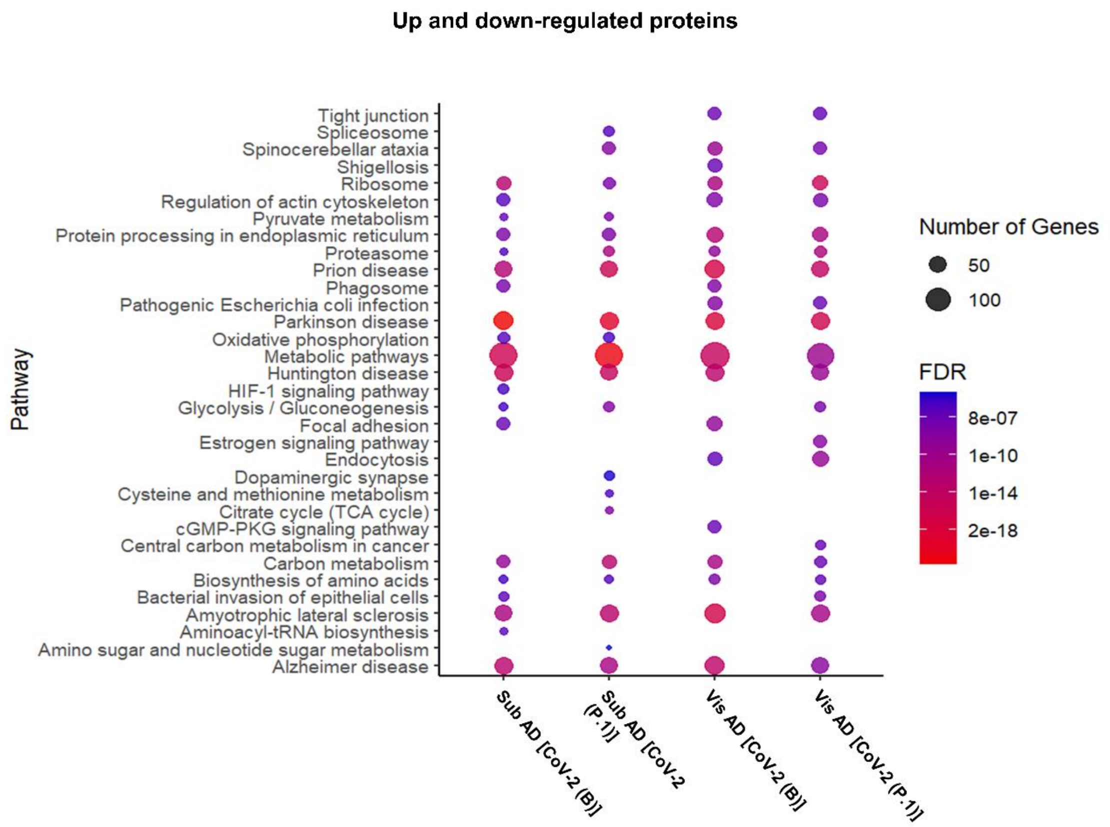

Supplementary Fig. 7. Comparative enrichment analysis of significantly altered proteins in the human adipose tissue cell proteome upon SARS-CoV-2 infection. Human adipose tissue-derived stromal-vascular cells isolated from subcutaneous adipose tissue or visceral adipose tissue were differentiated into adipocytes (Sub AD and Vis AD, respectively), exposed to the ancient [CoV-2(B)] or the P.1 [CoV-2(P.1)] SARS-CoV-2 lineages (MOI = 1 for 1h) and harvested 24 hours post-infection for proteomics analysis. The top 15 most enriched terms in the KEGG and Reactome databases for each comparison were combined and the number of differentially abundant proteins included in each term ("number of genes") and the FDR of the enrichment were compared. Proteomics were run using samples from 2-3 donors in technical triplicate. The mass spectrometry analysis was repeated once with similar results. Source data are provided in Supplementary Data 2.

Supplementary Table 1. Donor characteristics of post-mortem study.

Patients		*n (%)*
Total patients		47 (100)
	SARS-CoV-2 detection in adipose tissue	23 (49)
Sex		
	Females	23 (49)
	Males	24 (51)
Characteristics		***Value***
Age (in years)		
	Mean	65
	Median	65
	Range	38-88
	SD	14
Weight (in kg)		
	Mean	89
	Median	80
	Range	54.5-164
	SD	27
Height (in m)		
	Mean	1.68
	Median	1.67
	Range	1.44-1.89
	SD	0.10
BMI (in kg/m^2)		
	Mean	31.5
	Median	28.7
	Range	21.3-55.4
	SD	8.8

BMI, body mass index

SD, standard deviation

Supplementary Table 2. Primers and probes used in the study.

Gene	Oligos	Sequence
E	*Forward*	ACAGGTACGTTAATAGTTAATAGCGT
	Reverse	ATATTGCAGCAGTACGCACACA
	Probe	5’-FAM- ACACTAGCCATCCTTACTGCGCTTCG-BHQ1-3’ or 5’FAM-ACA CTA GCC ATC CTT ACT GCG CTT CG-QSY-3’
N2	*Forward*	TTACAAACATTGGCCGCAAA
	Reverse	GCGCGACATTCCGAAGAA
	Probe	5’-FAM-ACAATTTGCCCCCAGCGCTTCAG-BHQ1-3’
β-actin	*Forward*	CCCAGCCATGTACGTTGCTA
	Reverse	TCACCGGAGTCCATCACGAT
	Probe	5’-FAM-ACGCCTCTGGCCGTACCACTGG-TAMRA-3’
RNase P	*Forward*	AGATTTGGACCTGCGAGCG
	Reverse	GAGCGGCTGTCTCCACAAGT
	Probe	5’-FAM-TTCTGACCTGAAGGCTCTGCGCG-BHQ1-3’
FABP	*Forward*	CATGTGCAGAAATGGGATGGA
	Reverse	CGAACTTCAGTCCAGGTCAAC
LEP	*Forward*	TCTTCCTGCAAGGACTACGTT
	Reverse	GCCTTTGGAAGAGTGGCTTAG
INFA	*Forward*	GACTCCATCTTGGCTGTGA
	Reverse	TGATTTCTGCTCTGACAACCT
INFB	*Forward*	AAACTCATGAGCAGTCTGCA
	Reverse	AGGAGATCTTCAGTTTCGGAGG
IL6	*Forward*	GGTACATCCTCGACGGCATCT
	Reverse	GCCTCTTTGCTGCTTTCAC
IL1B	*Forward*	TCTTCGAGGCACAAGGCACA
	Reverse	GGCTGCTTCAGACACTTGAGC
TNFA	*Forward*	CCGAGGCAGTCAGATCATCTT
	Reverse	AGCTGCCCCTCAGCTTGA
CCL2	*Forward*	AGAATCACCAGCAGCAAGTGTCC
	Reverse	TCCTGAACCCACTTCTGCTTGG
GAPDH	*Forward*	AGGTCGGTGTGAACGGATTTG
	Reverse	TGTAGACCATGTAGTTGAGGTCA
	Forward	CAATGCTGCAATCGTGCTAC

CoV_2_N1	*Reverse*	GTTGCGACTACGTGATGAGG
ACE2	*Forward*	ACAGTCCACACTTGCCCAAAT
	Reverse	TGAGAGCACTGAAGACCCATT
NRP1	*Forward*	GCCACAGTGGAACAGGTGAT
	Reverse	ATGACCGTGGGCTTTTCTGT
TMPRSS2	*Forward*	CAAGTGCTCCAACTCTGGGAT
	Reverse	AACACACCGATTCTCGTCCTC
