# Supplementary material for: SARS-CoV-2 infects adipose tissue in a fat depot- and viral lineage-dependent manner
Source: Nat Commun. 2022 Sep 29;13:5722. doi: 10.1038/s41467-022-33218-8 (PMC9521555; doi:10.1038/s41467-022-33218-8)
Supplement: Supplementary file 3 — Description of Additional Supplementary Files [file 41467_2022_33218_MOESM3_ESM.pdf]

Description of Additional Supplementary Files

File Name: Supplementary Data 1
Description: Proteome data of human mesenchymal stem cells differentiated into adipocytes and infected with SARS-CoV-2 (24 hpi).

File Name: Supplementary Data 2
Description: Enriched terms of the KEGG and Reactome databases among the differentially expressed proteins comparing mock and SARS-CoV-2 infected fat cells.

File Name: Supplementary Data 3
Description: Reagents and materials used in the study
