# Supplementary material for: SARS-CoV-2 infects adipose tissue in a fat depot- and viral lineage-dependent manner
Source: Nat Commun. 2022 Sep 29;13:5722. doi: 10.1038/s41467-022-33218-8 (PMC9521555; doi:10.1038/s41467-022-33218-8)
Supplement: Supplementary file 7 — Reporting Summary [file 41467_2022_33218_MOESM7_ESM.pdf]

nature portfolio

Corresponding author(s): Daniel Martins-de-Souza, Mariana Kiomy Osako, Luiz Osório Leiria, and Marcelo A. Mori

Last updated by author(s): Aug 8, 2022

Reporting Summary

Nature Portfolio wishes to improve the reproducibility of the work that we publish. This form provides structure for consistency and transparency in reporting. For further information on Nature Portfolio policies, see our Editorial Policies and the Editorial Policy Checklist.

Statistics

For all statistical analyses, confirm that the following items are present in the figure legend, table legend, main text, or Methods section.

n/a	Confirmed	
☐	☒	The exact sample size (*n*) for each experimental group/condition, given as a discrete number and unit of measurement
☐	☒	A statement on whether measurements were taken from distinct samples or whether the same sample was measured repeatedly
☐	☒	The statistical test(s) used AND whether they are one- or two-sided<br>*Only common tests should be described solely by name; describe more complex techniques in the Methods section.*
☐	☒	A description of all covariates tested
☐	☒	A description of any assumptions or corrections, such as tests of normality and adjustment for multiple comparisons
☐	☒	A full description of the statistical parameters including central tendency (e.g. means) or other basic estimates (e.g. regression coefficient) AND variation (e.g. standard deviation) or associated estimates of uncertainty (e.g. confidence intervals)
☐	☒	For null hypothesis testing, the test statistic (e.g. *F*, *t*, *r*) with confidence intervals, effect sizes, degrees of freedom and *P* value noted<br>*Give P values as exact values whenever suitable.*
☒	☐	For Bayesian analysis, information on the choice of priors and Markov chain Monte Carlo settings
☒	☐	For hierarchical and complex designs, identification of the appropriate level for tests and full reporting of outcomes
☐	☒	Estimates of effect sizes (e.g. Cohen's *d*, Pearson's *r*), indicating how they were calculated

Our web collection on statistics for biologists contains articles on many of the points above.

Software and code

Policy information about availability of computer code

Data collection	MassLynx v4.1 was used to collect LC-MS/MS spectra. Bio-Rad CFX Manager was used to collect real-time qPCR data.
Data analysis	Progenesis QI for proteomics (Waters Corporation). For systems biology in silico: Cytoscape V3.9.0, ClusterProfiler R package V.4.0.3, Kyoto Encyclopedia of Genes and Genomes (KEGG) (release 102.0, April 1, 2022), Reactome (Accessed in June of 2022) and Metascape 3.5. Graph Prism 8.0 for data analysis and visualization. Image J for image analysis. For single-nucleus analysis: Cellranger and Seurat 4 package. These softwares were duly referenced in the article.

For manuscripts utilizing custom algorithms or software that are central to the research but not yet described in published literature, software must be made available to editors and reviewers. We strongly encourage code deposition in a community repository (e.g. GitHub). See the Nature Portfolio guidelines for submitting code & software for further information.

Data

Policy information about availability of data

All manuscripts must include a data availability statement. This statement should provide the following information, where applicable:

- Accession codes, unique identifiers, or web links for publicly available datasets
- A description of any restrictions on data availability
- For clinical datasets or third party data, please ensure that the statement adheres to our policy

All data generated or analysed during this study are included in this published article (and its supplementary information files) or in public repositories. The

proteomics data have been deposited at the ProteomeXchange Consortium via the PRIDE partner repository with the dataset identifier PXD034510 and DOI: 10.6019/PXD034510. Uniprot Homo sapiens reviewed proteome database (released 2022-03) was used for tandem mass spectra analysis. Single-nucleus RNA sequencing data used in this study is available in the ArrayExpress database with the dataset identifier E-MTAB-8564 (https://www.ebi.ac.uk/arrayexpress/experiments/E-MTAB-8564/). Donor information is presented in Supplementary Table 1 as averages to avoid individual identification. Individual data are protected and are not available due to data privacy laws. Source data are provided with this paper.

Human research participants

Policy information about studies involving human research participants and Sex and Gender in Research.

Reporting on sex and gender

For the postmortem sample analyses, we collected thoracic adipose tissue samples from 47 donors: 24 males and 23 females, who died from COVID-19 at the Clinics Hospital of Ribeirao Preto Medical School, SP, Brazil. Our data show that the viral load in thoracic adipose tissue is not associated with sex, and findings apply to both sexes.

For human adipose tissue stromal-vascular cell analyses, we isolated cells from abdominal subcutaneous adipose tissue and visceral omental adipose tissue of one male that underwent cholecystectomy and two females that underwent bariatric surgery at the Clinics Hospital of the University of Campinas, Campinas, SP, Brazil. Due to the limited number of samples, we could not test the hypothesis if the data generated using the isolated cells is influenced by sex.

Gender was not considered nor evaluated in our study, as this information has not been collected.

Population characteristics

We collected post-mortem samples from individuals aged 41 to 88 y (mean of 65 y), a mean height of 1.68 meters, a mean weight of 88 kg, and a mean BMI of 31. All 47 individuals died from COVID-19. We also used samples from one non-infected individual as a control.

We isolated human adipose tissue stromal-vascular cells from 3 donors aged 33, 43 and 46 (mean of 41 y), with BMI of 26, 34 and 35 (mean of 32). Donors were not diabetic. One of them had nonalcoholic steatohepatitis.

Recruitment

For the post-mortem samples, consecutive minimally invasive autopsies were performed following medical request as a routine procedure to clarify the cause of death of patients who died of COVID-19. We used sequential cases whose individuals had adipose tissue collected and stored at the local death verification service of the Clinics Hospital of Ribeirão Preto Medical School from May to July of 2020. Written informed consent was waived.

For human adipose tissue stromal-vascular cell isolation, individuals were approached by the investigators during care routine at the Clinics Hospital of the University of Campinas outpatient clinic before being called for the first consultation with the program professionals who ended up recruiting them to cholecystectomy or bariatric surgery. At that moment, they received written and oral information about the study before giving written informed consent to biopsy collection and use of the tissues. Inclusion criteria for individuals undergoing cholecystectomy were: BMI ≤ 24.9, age between 18 and 65 years. Inclusion criteria for individuals undergoing bariatric surgery were: BMI ≥ 35, age between 18 and 65 years, who achieved weight loss of at least 20% prior to the surgery from the moment of admission to the program until the return for surgical evaluation. Exclusion criteria were: significant intellectual limitation without adequate family support, uncontrolled psychiatric disorder, use of alcohol or illicit drugs, severe and decompensated cardiopulmonary disease, portal hypertension with esophagogastric varices, immunological or inflammatory diseases of the upper digestive tract that might have predisposed the individual to digestive bleeding or other risk conditions, Cushing's syndrome due to untreated adrenal hyperplasia or endocrine tumors, chronic liver disease, current or recent biliary obstruction (<6 months), acquired immunodeficiency (HIV), coagulopathies, or confirmed previous diagnosis of liver cirrhosis.

There is no perceived self-selection bias or other biases during recruitment that may impact the results. However, for some experiments we had to select samples based on their availability and quality. Immunofluorescence was conducted in post-mortem samples that had their morphology minimally preserved. The different human adipose tissue stromal-vascular cell pools used in this study were selected based on the quantity of cells and if we had enough cells from both subcutaneous and visceral depots available. These selections could have introduced bias to the study considering that quantity of samples may be related to the initial volume of the biopsy or the replicative capacity of the cells. On the other hand, post-mortem samples with preserved morphology could be more frequent in patients with less severe COVID-19 or milder adipose tissue infection. We believe these biases are unlikely to influence the main conclusions of the study, although the influence of human adipose tissue stromal-vascular cell replication on SARS-CoV-2 infection and the degree of adipose tissue damage in COVID-19 patients are yet to be investigated.

Ethics oversight

The protocols for human sample collection, storage, and analysis were conducted according to the guidelines of the Declaration of Helsinki and approved by the Institutional Research Ethics Committees of the University of São Paulo and University of Campinas (CAAE 48836721.3.0000.5440 and CAAE 78577417.8.0000.5404). The latter was confirmed by the National Ethics and Research Council.

Note that full information on the approval of the study protocol must also be provided in the manuscript.

Field-specific reporting

Please select the one below that is the best fit for your research. If you are not sure, read the appropriate sections before making your selection.

☒ Life sciences ☐ Behavioural & social sciences ☐ Ecological, evolutionary & environmental sciences

For a reference copy of the document with all sections, see nature.com/documents/nr-reporting-summary-flat.pdf

Life sciences study design

All studies must disclose on these points even when the disclosure is negative.

Sample size	Sample size was not predetermined. We were based on sample availability to determine the sample sizes in this study. For the post-mortem analyzes, sample size is larger than in other studies that evaluated similar parameters. For the human adipose tissue stromal-vascular cell analyses, we tried to minimize interindividual variability by using subcutaneous and visceral fat cells isolated from the same individuals. We have also performed experiments using independent pools of cells from the same donor or in technical replicates whenever possible to reduce experimental variability.
Data exclusions	Data were not excluded from the analyses, except for a few qPCR replicates when the results did not pass quality control (e.g., in case of selective evaporation or presence of bubbles in individual wells, or atypical amplification plot or melting curve when compared to the expected profile based on preoptimized conditions).
Replication	Experiments were run comparing independent pools of cells from the same donor and in technical replicates whenever possible to account for biological as well as technical variability. Key experiments were reproduced and all attempts at replication were successful. Experiments in Figures 1 and 2, Supplementary Figures 1, 4, 5, 6 and 7, and Supplementary Data 1 and 2 were repeated once. Experiments in Figure 3 were repeated once, with the exception of c-e. Experiments in Figure 4 were repeated once, with the exception of e and f. Experiments in Figure 5 were not repeated. Experiments in Supplementary Figure 2 were repeated at least once, with the exception of d and f-i. Experiments of Supplementary Figure 3c were not repeated. The experiments that were not repeated had low interassay variability, their controls worked as expected and biological and technical variability was accounted for in the analysis. Moreover, in most cases, their main findings were corroborated by other experiments. Information about reproducibility was included in the figure legends.
Randomization	Post-mortem samples were collected from people who died of COVID-19. All samples were treated as such. One non-infected individual was used as a control for the immunofluorescence assays. Samples were classified according to detection of viral RNA in adipose tissue and stratified according to sex and BMI. Viral load was also correlated with age, body weight and BMI.<br>Independent pools of differentiated human adipose tissue stromal-vascular cells were randomly split in two groups: mock controls and SARS-CoV-2-infected. Samples were analyzed in a random order.
Blinding	Sample collection, sample processing and data analysis were performed by different investigators. During this process, sample coding tried to avoid group identification during data collection. Only during data analysis information about group allocation was made available. For western blotting analyzes, samples were identified when they were injected in the gel to allow grouping. Nonetheless, band densitometry and quantification were performed blindly. Proteomic analyses were blinded for the near entirety of the workflow. First, samples were prepared, digested, and injected based on sample ID numbers that were not defined to the experimenters carrying out the analysis. Subsequently, all samples were automatically aligned and normalized to all proteins in Progenesis QI for Proteomics with no user input. Following these steps, the samples were then identified only by group. All posterior in silico analyses were performed after global filtering using preset cutoffs.

Reporting for specific materials, systems and methods

We require information from authors about some types of materials, experimental systems and methods used in many studies. Here, indicate whether each material, system or method listed is relevant to your study. If you are not sure if a list item applies to your research, read the appropriate section before selecting a response.

Materials & experimental systems

n/a	Involved in the study	
☐	☒	Antibodies
☐	☒	Eukaryotic cell lines
☒	☐	Palaeontology and archaeology
☒	☐	Animals and other organisms
☒	☐	Clinical data
☒	☐	Dual use research of concern

Methods

n/a	Involved in the study	
☒	☐	ChIP-seq
☒	☐	Flow cytometry
☒	☐	MRI-based neuroimaging

Antibodies

Antibodies used	1. Alpaca anti-mouse AlexaFluor 488 antibody

Antibodies used

Dilution: 1:1,000. According to the manufacturer (https://www.jacksonimmuno.com/catalog/products/615-545-214)
Supplier name: Jackson ImmunoReseacher
Catalog number: 615-545-214
Clone: polyclonal

2. Alpaca anti-rabbit AlexaFluor 594 antibody
Dilution: 1:1,000. According to the manufacturer (https://www.jacksonimmuno.com/catalog/products/611-585-215)
Supplier name: Jackson ImmunoReseacher
Catalog number: 611-585-215
Clone: polyclonal

3. Donkey anti-goat IgG AlexaFluor 488 antibody
Dilution: 1:800. According to the manufacturer (https://www.abcam.com/donkey-goat-igg-hl-alexa-fluor-488-ab150129.html)
Supplier name: Abcam
Catalog number: ab150129
Clone: polyclonal

4. Horse Anti-mouse IgG, HRP-linked Antibody
Dilution: 1:10,000
Supplier name: Cell Signaling Technology
Catalog number: 7076
Clone: polyclonal

5. Goat Anti-rabbit IgG, HRP-linked Antibody
Dilution: 1:10,000
Supplier name: Cell Signaling Technology
Catalog number: 7074
Clone: polyclonal

6. Donkey anti-Mouse IgG (H+L) Highly Cross-Adsorbed Secondary Antibody, Alexa Fluor 488
Dilution: 1:800
Supplier name: Thermo Fisher Scientific
Catalog number: A21202
Clone: polyclonal

7. Goat Anti-Mouse IgG H&L (Alexa Fluor® 594)
Dilution: 1:800
Supplier name: Abcam
Catalog number: ab150116
Clone: polyclonal

8. Goat anti-rabbit IgG AlexaFluor 405 antibody
Dilution: 1:800. According to manufacturer (https://www.abcam.com/goat-rabbit-igg-hl-alexa-fluor-405-ab175652.html)
Supplier name: Abcam
Catalog number: ab175652
Clone: polyclonal

9. Goat polyclonal anti-ACE2 antibody
Dilution: 1:200
Supplier name: R&D system
Catalog number: AF933
Clone: polyclonal

10. Mouse monoclonal anti-ACE-2 antibody
Dilution: 1:3,000. According to manufacturer's suggestion 1:2,000- 1:10,000 (https://www.novusbio.com/products/ace-2-antibody-ac18f_nbp2-80035)
Supplier name: Novus Biological
Catalog number: NBP2-80035
Clone: AC18F

11. Mouse monoclonal anti-double stranded(ds) RNA antibody
Dilution: 1:1,000
Supplier name: SCICONS
Catalog number: 10010200
Clone: J2

12. Mouse monoclonal anti-perilipin 1 (G-2)
Dilution: 1:50
Supplier name: Santa Cruz Biotechnology
Catalog number: sc-390169
Clone: G-2

13. Mouse monoclonal anti-Vinculin antibody
Dilution: 1:2,000. According to manufacturer suggestion 1:1,000- 1:2,000 (https://www.abcam.com/vinculin-antibody-epr19579-ab207440.html?productWallTab=ShowAll)
Supplier name: Abcam

Catalog number: ab207440
Clone: EPR19579

14. Rabbit monoclonal Neuropilin-1 antibody
Dilution: 1:200
Supplier name: Laboratory of Precision and Nanomedicine, Institute of Biomedicine and Translational Medicine, University of Tartu
Catalog number: n/d
Clone: 3E7

15. Rabbit monoclonal Neuropilin-1 antibody
Dilution: 1:200
Supplier name: Laboratory of Precision and Nanomedicine, Institute of Biomedicine and Translational Medicine, University of Tartu
Catalog number: n/d
Clone: 3E8

16. Rabbit monoclonal anti-SARS-CoV-2 Spike antibody
Dilution: 1:500
Supplier name: Invitrogen
Catalog number: 703959
Clone: T01KHuRb

17. Rabbit polyclonal anti-ADFP (perilipin 2)
Dilution: 1:100
Supplier name: Abcam
Catalog number: ab78920
Clone: polyclonal

18. Rabbit polyclonal anti-ATGL
Dilution: 1:1,000
Supplier name: Cell Signaling Technology
Catalog number: 2138
Clone: polyclonal

19. Rabbit polyclonal anti-HSL
Dilution: 1:1,000
Supplier name: Cell Signaling Technology
Catalog number: 4107
Clone: polyclonal

20. Rabbit polyclonal anti-phospho-HSL(S565)
Dilution: 1:1,000
Supplier name: Cell Signaling Technology
Catalog number: 4137
Clone: polyclonal

21. Rabbit polyclonal anti-SARS-CoV-2 Spike
Dilution: 1:100
Supplier name: Rheabiotech
Catalog number: IM-0828
Clone: polyclonal

22. Rabbit polyclonal anti-α-tubulin antibody
Dilution: 1:5,000
Supplier name: Cell Signaling Technology
Catalog number: 2144
Clone: polyclonal

Validation

Antibody validation was provided in the manuscript, in previous studies and/or by the vendor. Secondary antibody controls (secondary antibody incubation in the absence of primary antibody) and autofluorescence controls were conducted for immunofluorescence assays. Uncropped blots were also presented to show band sizes and number of bands, showing overall unique bands at the expected sizes. Below is information about further validation for the antibodies used in this study:

1. Goat polyclonal anti-ACE2 antibody
Supplier name: R&D system
Catalog number: AF933
Validation: According to Lee et al 2020. The authors evaluated ACE2 expression compared to isotype control in human tracheal tissue (https://www.nature.com/articles/s41467-020-19145-6);

2. Mouse monoclonal anti-ACE-2 antibody
Supplier name: Novus Biological
Catalog number: NBP2-80035
Validation: According to the manufacturer (https://www.novusbio.com/products/ace-2-antibody-ac18f_nbp2-80035). The supplier datasheet shows Western Blot for ACE-2 Antibody (AC18F) [NBP2-80035] - Western blot analysis using anti-ACE2 (human), mAb (AC18F) at 1:2,000 dilution. Detection of hACE2 (Fc protein and Ecto domain (FLAG(R)-tagged)). Also, we used Caco-2 protein extract to confirm ACE2 detection in a cell known to express it.

3. Mouse monoclonal anti-double stranded(ds) RNA antibody
Supplier name: SCICONS
Catalog number: 10010200
Validation: According to Veras et al, 2020. The authors evaluated dsRNA in SARS-CoV-2–infected or non-infected human neutrophils (https://rupress.org/jem/article/217/12/e20201129/152086/SARS-CoV-2-triggered-neutrophil-extracellular).

4. Mouse monoclonal anti-perilipin 1 (G-2)
Supplier name: Santa Cruz Biotechnology
Catalog number: sc-390169
Validation: According to the manufacturer, showing western blot analysis in non-transfected and human perilipin transfected HEK293T (https://www.scbt.com/p/perilipin-antibody-g-2?productCanUrl=perilipin-antibody-g-2&_requestid=1072310), and Sugihara et al. 2019 (https://rupress.org/jcb/article/218/3/949/120793/The-AAA-ATPase-ubiquitin-ligase-mysterin).

5. Mouse monoclonal anti-Vinculin antibody
Supplier name: Abcam
Catalog number: ab207440
Validation: According to manufacturer (https://www.abcam.com/vinculin-antibody-epr19579-ab207440.html?productWallTab=ShowAll); knockout validated: ab207440 Anti-Vinculin antibody [EPR19579] was shown to specifically react with vinculin in wild-type HeLa cells. Loss of signal was observed when knockout cell line ab265580 (knockout cell lysate ab257795) was used.

6. Rabbit monoclonal Neuropilin-1 antibody
Supplier name: Laboratory of Precision and Nanomedicine, Institute of Biomedicine and Translational Medicine, University of Tartu
Validation: According to Cantuti-Castelvetri et al., 2020 and Daly et al. 2020. The treatment of HEK-293T with anti-NRP1 significantly reduced infection by SARS-CoV-2 pseudoviruses in cells expressing NRP1 (Cantuti-Castelvetri et al, 2020; https://www.science.org/doi/10.1126/science.abd2985). Indeed, Daly and collaborators stained NRP1 by immunofluorescence in NRP1-expressing PPC-1 (human primary prostate cancer) cells but not in M21 (human melanoma) cells that do not express NRP1. Moreover, the authors shown reduced SARS-CoV-2 infection upon treatment with anti-NRP-1 compared to a control mAb targeting avian influenza A virus (H11N3) hemagglutinin in Caco-2 cells (Daly et al, 2020; https://www.science.org/doi/10.1126/science.abd3072).

7. Rabbit monoclonal Neuropilin-1 antibody
Supplier name: Laboratory of Precision and Nanomedicine, Institute of Biomedicine and Translational Medicine, University of Tartu
Validation: Validation: According to Cantuti-Castelvetri et al., 2020 and Daly et al. 2020. The treatment of HEK-293T with anti-NRP1 significantly reduced infection by SARS-CoV-2 pseudoviruses in cells expressing NRP1 (Cantuti-Castelvetri et al, 2020; https://www.science.org/doi/10.1126/science.abd2985). Indeed, Daly and collaborators stained NRP1 by immunofluorescence in NRP1-expressing PPC-1 (human primary prostate cancer) cells but not in M21 (human melanoma) cells that do not express NRP1. Moreover, the authors shown reduced SARS-CoV-2 infection upon treatment with anti-NRP-1 compared to a control mAb targeting avian influenza A virus (H11N3) hemagglutinin in Caco-2 cells (Daly et al, 2020; https://www.science.org/doi/10.1126/science.abd3072).

8. Rabbit monoclonal anti-SARS-CoV-2 Spike antibody
Supplier name: Invitrogen
Catalog number: 703959
Validation: According to manufacturer (https://www.thermofisher.com/antibody/product/SARS-CoV-2-Spike-Protein-RBD-Chimeric-Antibody-clone-T01KHuRb-Recombinant-Monoclonal/703959); The supplier datasheet shows immunofluorescence of HEK-293 cells transfected with a vector encoding full length spike protein from SARS-CoV2 compared with untransfected cells.

9. Rabbit polyclonal anti-ADFP (perilipin 2)
Supplier name: Abcam
Catalog number: ab78920
Validation: According to the manufacturer's website, the antibody had its specificity confirmed by extensive validation (https://www.abcam.com/adfp-antibody-ab78920.html?productWallTab=ShowAll). We also relied on validation by Pillai, Smitha, et al. (https://doi.org/10.1016/j.celrep.2022.110796).

10. Rabbit polyclonal anti-ATGL
Supplier name: Cell Signaling Technology
Catalog number: 2138
Validation: According to the manufacturer (https://www.cellsignal.com/products/primary-antibodies/atgl-antibody/2138).The supplier datasheet shows western blot analysis of extracts from NIH/3T3 and differentiated NIH/3T3-L1 cells, using ATGL antibody.

11. Rabbit polyclonal anti-HSL
Supplier name: Cell Signaling Technology
Catalog number: 4107
Validation: According to the manufacturer (https://www.cellsignal.com/products/primary-antibodies/hsl-antibody/4107). The supplier datasheet shows western blot analysis of extracts from NIH/3T3 and 3T3-L1 cells using HSL Antibody.

12. Rabbit polyclonal anti-phospho-HSL(S565)
Supplier name: Cell Signaling Technology
Catalog number: 4137
Validation: According to the manufacturer (https://www.cellsignal.com/products/primary-antibodies/phospho-hsl-ser565-antibody/4137). The supplier datasheet shows western blot analysis of extracts from differentiated 3T3-L1 cells treated with forskolin, oligomycin or lambda protein phosphatase using Phospho-HSL (Ser565) antibody.

13. Rabbit polyclonal anti-SARS-CoV-2 Spike
Supplier name: Rheabiotech

Catalog number: IM-0828
Validation: Antibody specificity for immunofluorescence was validated by Crunfli et al. (https://www.medrxiv.org/content/10.1101/2020.10.09.20207464v5) using SARS-CoV-2-infected Vero cells and brain slices.

14. Rabbit polyclonal anti-α-tubulin antibody
Supplier name: Cell Signaling Technology
Catalog number: 2144
Validation: The manufacturer's website present various examples of antibody validation (https://www.cellsignal.com/products/primary-antibodies/a-tubulin-antibody/2144). This antibody has been used in a number of studies, including this one: https://www.nature.com/articles/s41467-022-31899-9.

Eukaryotic cell lines

Policy information about cell lines and Sex and Gender in Research

Cell line source(s)	Vero CCL-81 and P3X63Ag8.653 cells were obtained from ATCC. Human primary adipose tissue stromal-vascular cells were isolated from abdominal subcutaneous adipose tissue and visceral omental adipose tissue of one male that underwent cholecystectomy and two females that underwent bariatric surgery.
Authentication	Cells were not authenticated, but Vero CCL-81 cells behaved as expected both morphologically as well as for their permissiveness to SARS-CoV-2 infection and replication. P3X63Ag8.653 cells also behaved as expected morphologically and in their capacity to allow hybridoma formation and antibody production.
Mycoplasma contamination	Cells were not tested for mycoplasma contamination.
Commonly misidentified lines (See ICLAC register)	No misidentified lines were used.
